# Real-World Predictors of Survival in CDK4/6 Inhibitor-Treated Metastatic Breast Cancer: The Significance of ER Expression Level and Treatment Naivety

**DOI:** 10.3390/curroncol32120709

**Published:** 2025-12-16

**Authors:** Büşra Bülbül, Bekir Ucun, Can Cangür, İrem Turgut Yeğen, Orhan Önder Eren, Cengiz Yılmaz, Gürkan Gül, Atike Pınar Erdoğan, Ece Şahin Hafızoğlu, Erhan Gökmen, Oguzcan Ozkan, Murat Araz, Ahmet Oruç, Serkan Yıldırım

**Affiliations:** 1 Medical Oncology Department Tıbbi Onkoloji, Medical Faculty, Selçuk University, Konya 42250, Turkeycan.cangur@selcuk.edu.tr (C.C.); irem.turgutyegen@selcuk.edu.tr (İ.T.Y.);; 2 Medical Oncology, İzmir City Hospital, İzmir 35540, Turkey; 3 Medical Oncology Department, Medical Faculty, Manisa Celal Bayar University, Manisa 45030, Turkey; 4 Medical Oncology Department, Medical Faculty, Ege University, İzmir 35100, Turkey; 5 Medical Oncology Department, Medical Faculty, Necmettin Erbakan University, Konya 42090, Turkey

**Keywords:** breast cancer, CDK4/6 inhibitors, oestrogen receptor, high ER expression, treatment naivety, overall survival, real-world evidence, biomarker

## Abstract

**Simple Summary:**

CDK4/6 inhibitors represent a cornerstone in managing hormone receptor-positive, HER2-negative metastatic breast cancer. However, identifying which patients derive the greatest benefit remains challenging. In this multicenter analysis of 603 patients, we demonstrate that treatment-naive patients and those with exceptionally high oestrogen receptor (ER) expression (≥90%) experience significantly longer overall survival when treated with CDK4/6 inhibitors. These findings provide clinicians with practical biomarkers for optimizing treatment selection and highlight the importance of quantitative ER assessment beyond conventional positivity thresholds.

**Abstract:**

Objective: CDK4/6 inhibitors constitute standard first-line therapy for hormone receptor (HR)-positive, HER2-negative metastatic breast cancer (MBC). We investigated real-world predictors of overall survival (OS), with particular focus on high ER expression (≥90%). Methods: In this multicenter, retrospective study, we analyzed 603 HR-positive/HER2-negative MBC patients treated with CDK4/6 inhibitors (ribociclib or palbociclib) between May 2020 and June 2024. We evaluated demographic, clinical, and pathological factors for their impact on OS using univariate and multivariate Cox regression analyses. Results: In univariate analysis, significantly longer OS was observed in endocrine therapy-naive patients (median OS: 51.0 vs. 33.3 months; *p* < 0.001), those without liver metastases (50.0 vs. 34.0 months; *p* = 0.019), bone-only metastases (57.7 vs. 40.5 months; *p* = 0.022), and PR-positive patients (50.0 vs. 36.0 months; *p* = 0.037). Patients with ER expression ≥90% showed a strong trend toward longer OS (49.0 vs. 41.0 months; *p* = 0.072). In multivariate analysis, endocrine therapy naivety (*p* = 0.045) and high ER expression (≥90%) (*p* = 0.031) emerged as independent predictors of superior OS. Conclusions: Our study identifies treatment naivety and exceptionally high ER expression (≥90%) as key independent predictors of prolonged OS in CDK4/6 inhibitor-treated MBC patients. These findings underscore the importance of early CDK4/6 inhibitor implementation and suggest that quantitative ER assessment may refine patient selection beyond conventional positivity thresholds.

## 1. Introduction

Breast cancer remains the most frequently diagnosed malignancy and the leading cause of cancer-related mortality among women worldwide [1]. Hormone receptor (HR)-positive, HER2-negative disease represents the most common molecular subtype of metastatic breast cancer (MBC), for which endocrine therapy has historically formed the therapeutic backbone [2].

The introduction of cyclin-dependent kinase 4/6 (CDK4/6) inhibitors has substantially improved outcomes for patients with HR-positive/HER2-negative MBC. When combined with endocrine therapy, these agents have demonstrated significant improvements in both progression-free survival (PFS) and overall survival (OS) across multiple phase III trials, establishing them as the first-line standard of care per current international guidelines [3].

The efficacy of CDK4/6 inhibitors stems from their targeting of the cyclin D-CDK4/6-retinoblastoma (Rb) pathway, a critical downstream effector of oestrogen receptor (ER) signalling [4]. In ER-positive breast cancer, cyclin D-CDK4/6 complex formation promotes Rb phosphorylation, triggering cell cycle progression from the G1 phase to the S phase [5]. The synergistic inhibition of both ER signalling and CDK4/6 has proven highly effective in advanced breast cancer, forming the rational basis for their combined use [6].

Robust clinical evidence supports this approach. Ribociclib has demonstrated significant OS benefits in combination with endocrine therapy in both post-menopausal (MONALEESA-2) and pre-menopausal (MONALEESA-7) populations [7]. Similarly, palbociclib showed substantial PFS improvement with letrozole in the PALOMA-2 trial [8], with subsequent analyses confirming OS benefits in first-line settings [9]. Abemaciclib, with its distinct pharmacologic properties, also demonstrated significant PFS improvement when combined with a nonsteroidal aromatase inhibitor in the MONARCH 3 trial [10].

Despite these advances, significant heterogeneity in the treatment response persists. While CDK4/6 inhibitors combined with endocrine therapy represent the gold standard first-line treatment for HR-positive/HER2-negative MBC, acquired resistance develops in most patients, and certain subgroups derive limited benefit [11]. Consequently, identifying clinical and pathological biomarkers to predict which patients are most likely to benefit from CDK4/6 inhibitors represents an urgent unmet need.

The ER expression level represents a compelling candidate biomarker. While a 1% threshold defines ER positivity clinically, emerging evidence suggests a continuous relationship between ER abundance and therapeutic response [12]. Previous studies have indicated that higher ER expression levels correlate with improved outcomes with endocrine therapy alone [13], and subgroup analyses from pivotal CDK4/6 inhibitor trials suggest enhanced benefit in patients with higher ER expression [7,11]. However, these studies utilised varying thresholds, and the optimal cutoff for predicting CDK4/6 inhibitor benefit remains undefined. Quantitative ER expression is considered a potential surrogate for tumor endocrine sensitivity. We hypothesized that tumours exhibiting exceptionally high ER expression (≥90%) represent a subset with maximal endocrine sensitivity, which may, in turn, translate to a superior response to the synergistic blockade of both the ER pathway and the cell cycle via CDK4/6 inhibitors.

In this multi-centre real-world study, we investigated a comprehensive set of clinical and pathological parameters to identify predictors of OS in patients receiving CDK4/6 inhibitor therapy. Based on a biological rationale suggesting that tumours with exceptionally high ER expression might exhibit maximal dependence on the ER pathway, we specifically hypothesised that an ER expression threshold of ≥90% would identify patients deriving superior benefit from CDK4/6 inhibition.

## 2. Materials and Methods

### 2.1. Study Design and Patient Selection

**Figure 1:**
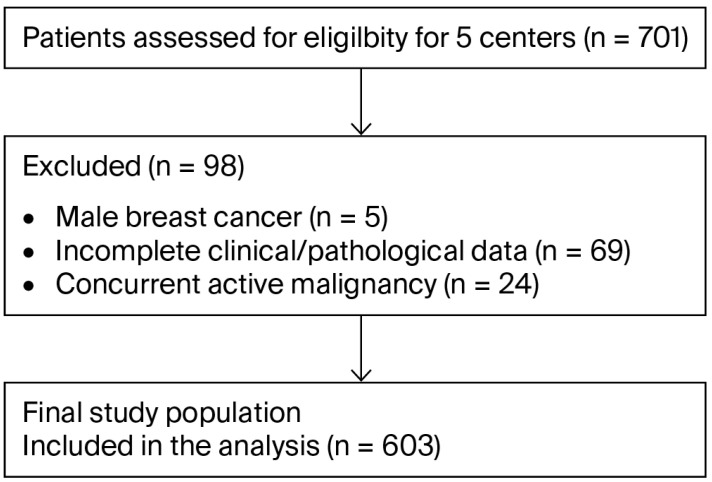
Patient selection flowchart.

This multicenter, retrospective study analyzed data from consecutive patients with HR-positive/HER2-negative MBC treated with CDK4/6 inhibitors (ribociclib or palbociclib) at five tertiary medical centers in Turkey between May 2020 and June 2024. A flowchart illustrating patient selection is presented in Figure 1.

Inclusion criteria were: (1) histologically confirmed metastatic breast cancer; (2) HR-positive (ER ≥ 1% and/or PR ≥ 1%) and HER2-negative status; (3) documented pathology report; (4) treatment with a CDK4/6 inhibitor at any line of therapy; and (5) female sex.

Exclusion criteria included: (1) male breast cancer; (2) incomplete clinical or pathological data; and (3) concurrent active malignancy.

HER2 status was determined according to ASCO/CAP guidelines [10]. HER2-negative was defined as immunohistochemistry (IHC) 0 or 1+, or IHC 2+ with negative in situ hybridization. HER2-low was defined as IHC 1+ or IHC 2+ with negative in situ hybridization. HER2-positive cases (IHC 3+ or IHC 2+ with positive in situ hybridization) were excluded.

Endocrine therapy naivety was defined as no prior endocrine therapy for metastatic disease. Consequently, all non-naive patients (23.9%) initiated CDK4/6 inhibitors in the second or later line. Overall, the CDK4/6 inhibitor was started in the first line for 76.1% of the cohort and in subsequent lines for 23.9%.

### 2.2. Data Collection and Variables

We collected comprehensive demographic, clinical and pathological data, including: age at CDK4/6 inhibitor initiation, menopausal status, metastatic status (de novo vs. recurrent), metastatic sites (visceral, bone-only or liver), histological subtype, HER2 status (low vs. negative), Oestrogen (ER) expression level (<90% vs. ≥90%), Progesterone (PR) status (negative vs. positive), specific CDK4/6 inhibitor used (ribociclib vs. palbociclib) and prior endocrine therapy or chemotherapy before CDK4/6 inhibitor initiation.

An ER expression threshold of ≥90% was selected based on biological rationale and clinical considerations. Pre-clinical data suggest a continuous relationship between ER abundance and sensitivity to endocrine therapy [12]. Clinically, while lower thresholds define ER positivity, emerging evidence suggests that higher thresholds may better identify tumours with maximal endocrine dependence [13]. The ≥90% threshold was chosen to identify a population with very high ER expression that might derive exceptional benefit from CDK4/6 inhibitor therapy. This was a pre-specified analysis to test this specific hypothesis.

### 2.3. Outcomes and Statistical Analysis

The primary endpoint was OS, defined as the time from CDK4/6 inhibitor initiation to death from any cause. Patients alive at the last follow-up were censored.

Categorical variables are presented as frequencies and percentages, and continuous variables are presented as medians with ranges. Survival curves were estimated using the Kaplan–Meier method and compared with log-rank tests. Univariate Cox proportional hazards models were used to assess the impact of each variable on OS. Variables with a *p*-value < 0.1 in the univariate analysis were included in a multivariate Cox regression model to identify independent predictors. All statistical analyses were performed using SPSS version 20.0 (IBM Corp., Armonk, NY, USA), with statistical significance defined as *p* < 0.05.

### 2.4. Ethical Considerations

The study was conducted in accordance with the Declaration of Helsinki and approved by the Institutional Review Board of Selçuk University Faculty of Medicine (Protocol code: 2025/20, Date of approval: 14 January 2025). Patient consent was waived due to the retrospective nature of the study, and the analysis used anonymized clinical data.

## 3. Results

### 3.1. Patient Characteristics

A total of 603 patients met the inclusion criteria. Baseline characteristics are summarised in Table 1. The median age was 56.5 years (range 28–85). Most patients were post-menopausal (66.5%) and had invasive ductal carcinoma (87.1%). HER2-low status was present in 49.3% of the patients. Regarding ER expression, 310 patients (51.4%) had an ER ≥ 90%, while 259 (43.0%) had an ER < 90%. De novo metastatic disease was present in 51.1% of the patients. Visceral metastases were observed in 52.4% of the patients, liver metastases in 22.6% and bone-only metastases in 22.4%. Ribociclib was used more frequently than palbociclib (70.0% vs. 30.0%). Most patients were endocrine therapy-naive (76.0%) and chemotherapy-naive (69.3%) before initiating CDK4/6 inhibitor therapy.

### 3.2. Univariate Analysis of Overall Survival

**Figure 2:**
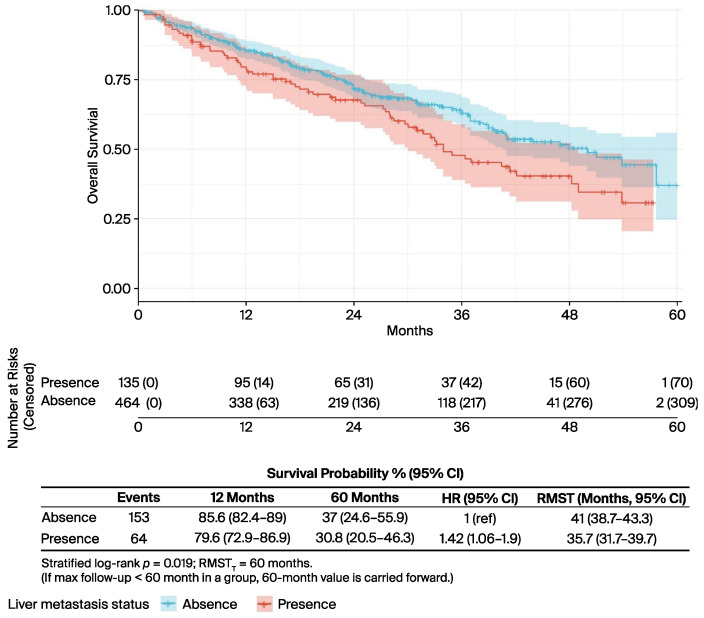
Liver metastasis status, univariate analysis. Kaplan–Meier curves for overall survival by metastatic pattern (liver metastasis vs. other sites). The risk table shows the number of patients at risk at specified time intervals. *p*-value from log-rank test is shown.

**Figure 3:**
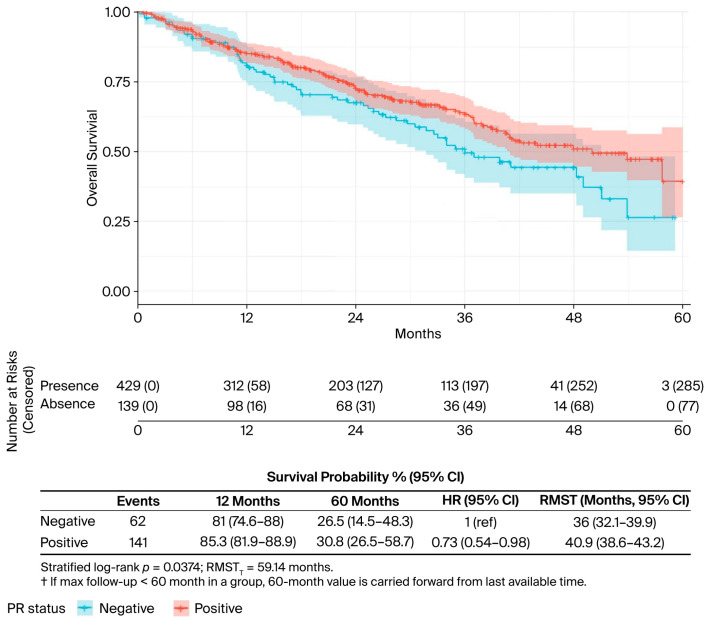
Only bone metastasis status, univariate analysis. Kaplan–Meier curves for overall survival by metastatic pattern (bone-only metastases vs. other sites). The risk table shows the number of patients at risk at specified time intervals. *p*-value from log-rank test is shown.

**Figure 4:**
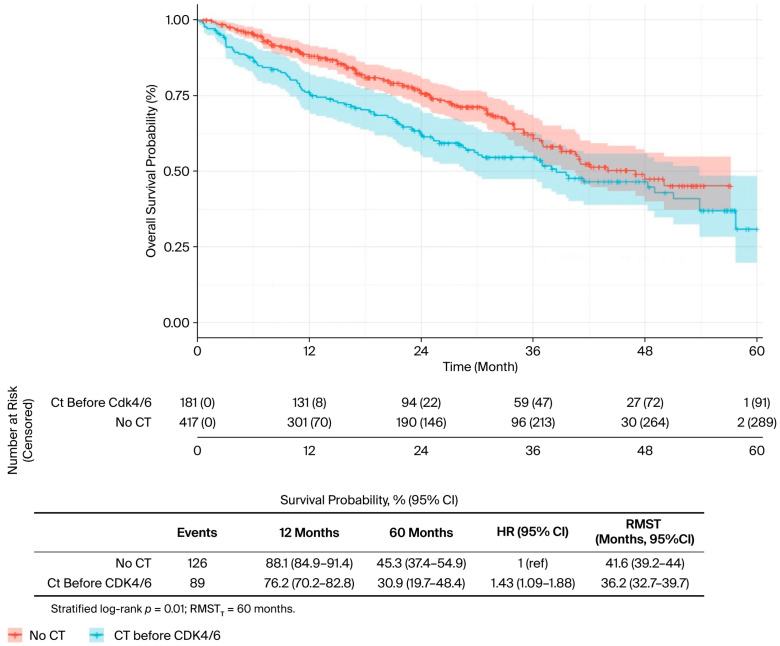
PR status, univariate analysis. Kaplan–Meier curves for overall survival by progesterone receptor (PR) status. The risk table shows the number of patients at risk at specified time intervals. *p*-value from log-rank test is shown.

**Figure 5:**
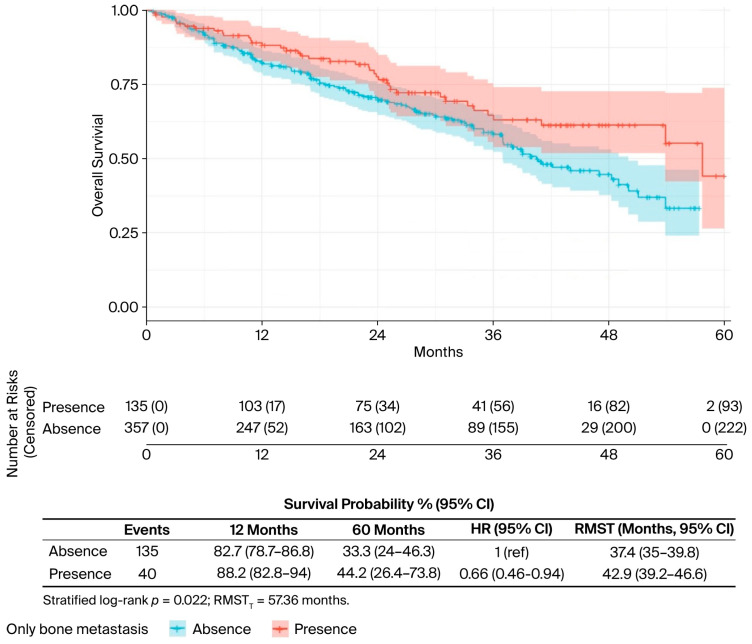
Endocrine therapy before CDK 4/6 Inhibitor, univariate analysis (ET: Endocrine therapy).

**Figure 6:**
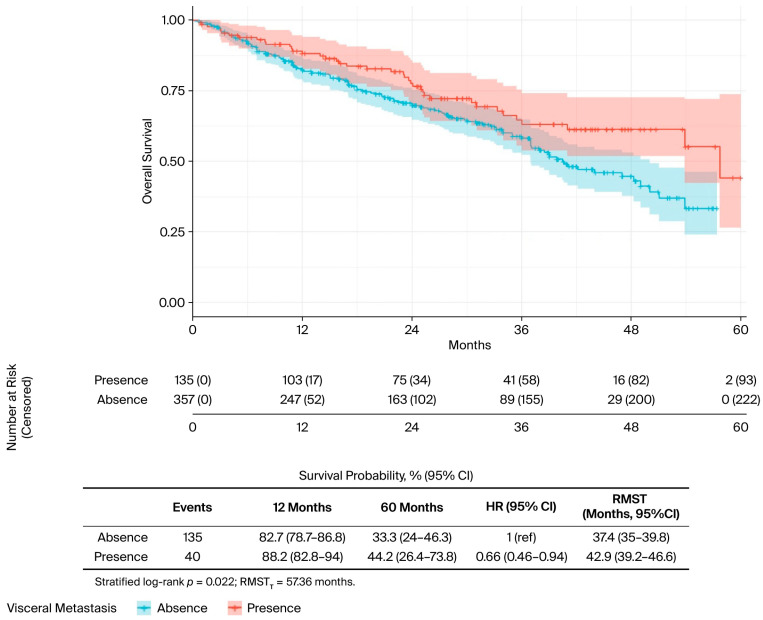
Chemotherapy before CDK 4/6 Inhibitors, univariate analysis (CT: Chemotherapy).

**Figure 7:**
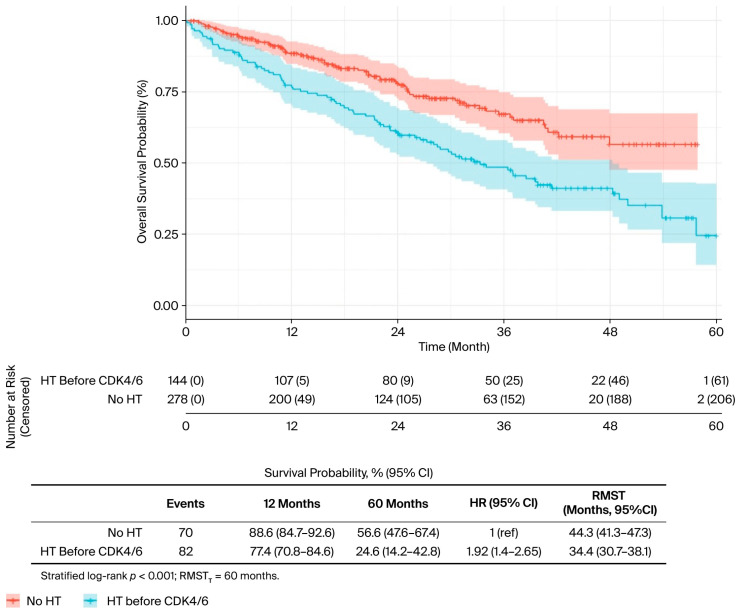
Visceral metastasis status, univariate analysis. Kaplan–Meier curves for overall survival by visceral metastasis status. The risk table shows the number of patients at risk at specified time intervals. *p*-value from log-rank test is shown.

**Figure 8:**
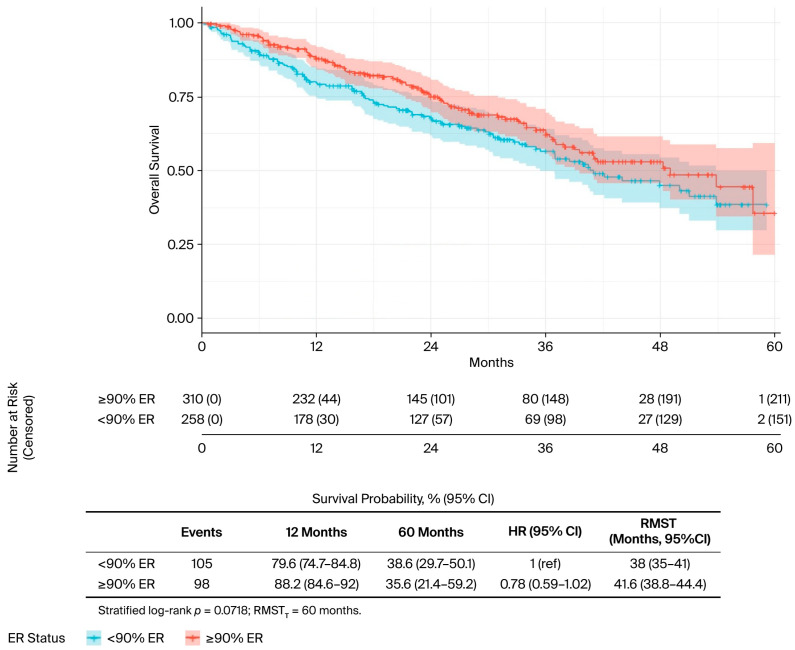
ER status, univariate analysis. Kaplan–Meier curves for overall survival by oestrogen receptor (ER) status. The risk table shows the number of patients at risk at specified time intervals. *p*-value from log-rank test is shown.

In the univariate analysis (Table 2), several factors were significantly associated with OS. The presence of liver metastases was associated with worse outcomes (median OS: 34.0 vs. 50.0 months; *p* = 0.019) (Figure 2), while bone-only metastases conferred a favourable prognosis (57.7 vs. 40.5 months; *p* = 0.022) (Figure 3). PR-positive status was associated with longer OS (50.0 vs. 36.0 months; *p* = 0.037) (Figure 4). Endocrine therapy-naive patients had substantially longer median OS compared to those with prior endocrine therapy exposure (51.0 vs. 33.3 months; *p* < 0.001) (Figure 5. Similarly, chemotherapy-naïve patients showed longer OS (47.0 vs. 38.5 months; *p* = 0.010) (Figure 6). Similarly, patients with visceral metastases had a significantly shorter median OS compared to those without (40.5 vs. 51.0 months; *p* = 0.025) (Figure 7). Although ER expression ≥90% showed only a trend towards significance in univariate analysis (49.0 vs. 41.0 months; *p* = 0.072) (Figure 8), it was included in the multivariate model based on our pre-specified hypothesis. No significant differences in OS were observed based on CDK4/6 inhibitor type, HER2 status, menopausal status, metastatic status or histological subtype.

### 3.3. Multivariate Analysis of Overall Survival

In the multivariate Cox regression analysis (Table 3), which included variables with *p* < 0.1 from the univariate analysis, two factors remained independently associated with superior OS: endocrine therapy naivety and high ER expression (≥90%) (HR: 0.74, 95% CI [0.56, 0.97]; *p* = 0.031). The presence of liver metastasis, which was significant in the univariate analysis, showed a numerical but not statistically significant association in the multivariate model (HR: 1.25, 95% CI [0.92, 1.70]; *p* = 0.157).

## 4. Discussion

This large multicentre real-world study identified two key independent predictors of prolonged overall survival in HR-positive/HER2-negative metastatic breast cancer patients treated with CDK4/6 inhibitors: naivety to endocrine therapy before CDK4/6 inhibitor initiation and exceptionally high ER expression (≥90%). These findings have important implications for clinical practice and patient selection.

Our most significant finding concerns the prognostic importance of quantitative ER expression. While ER positivity is traditionally defined by a ≥1% threshold, accumulating evidence suggests a continuous relationship between ER abundance and therapeutic response [10,12]. Previous studies have indicated that higher ER expression levels correlate with improved outcomes with endocrine therapy alone [12], and subgroup analyses from pivotal CDK4/6 inhibitor trials have suggested enhanced efficacy in patients with higher ER expression [7,11]. However, these investigations utilised varying thresholds, and to our knowledge, this is the first study to specifically demonstrate that an ER expression threshold of ≥90% identifies patients deriving superior overall survival benefit from CDK4/6 inhibitor therapy in a real-world setting.

The biological plausibility of this finding is substantial. Tumours with exceptionally high ER expression likely exhibit maximal dependence on the ER signalling pathway, rendering them particularly vulnerable to the combined ER pathway and CDK4/6 inhibition [5,6]. This heightened dependence may translate to more durable disease control and improved survival outcomes. Our data suggest that the ≥90% threshold may represent a clinically meaningful cutoff that could help identify patients most likely to achieve long-term benefit from first-line CDK4/6 inhibitor therapy.

The superior outcomes observed in endocrine therapy-naive patients align with the established biology of acquired endocrine resistance [13]. Patients who have progressed on prior endocrine therapy likely harbour resistance mechanisms that can diminish the effectiveness of subsequent CDK4/6 inhibition [12]. Our results strongly reinforce current guideline recommendations to implement CDK4/6 inhibitor combinations in first-line settings to maximise patient benefit [3]. The loss of statistical significance for prior chemotherapy in the multivariate analysis underscores that prior endocrine therapy status is the more dominant factor influencing OS in this treatment context.

Although PR positivity was associated with better OS in the univariate analysis, consistent with its role as a functional indicator of an intact ER pathway [13], it was not an independent factor in our multivariate model. This suggests that its prognostic information may be superseded by the more powerful effects of quantitative ER expression and prior treatment history. Similarly, the favourable prognosis associated with bone-only metastases [14,15] and the poor prognosis linked to liver metastases [16,17] observed in our univariate analysis are well documented. Their non-significance in the multivariate model indicates that their impact may be contextual and influenced by other stronger predictors.

Our findings regarding other clinical parameters are consistent with those of the broader literature. We found no significant difference in OS based on the specific CDK4/6 inhibitor used, which aligns with real-world comparative effectiveness studies [18]. Similarly, no significant differences were observed based on HER2-low status, supporting recent evidence that HER2-low and HER2-negative tumours derive similar benefit from CDK4/6 inhibitors [19,20,21].

The loss of independent prognostic significance for PR status and the presence of liver metastases in the multivariate model is noteworthy. This suggests that the prognostic information provided by PR positivity may be captured by the more dominant predictors of quantitative ER expression and prior treatment history. Similarly, the impact of established prognostic factors such as liver metastases may become contextual and less distinct when adjusted for the stronger driving forces of tumor biology and treatment line.

Several limitations warrant consideration. The retrospective design of this study introduces the potential for unmeasured confounding despite multivariate adjustment. Selection bias is possible, as patients receiving CDK4/6 inhibitors in real-world practice may differ from clinical trial populations. The heterogeneity in treatment protocols across five centres, while reflecting real-world practice, introduces variability. The lack of a central pathology review is another limitation, though all centres followed ASCO/CAP guidelines [10]. Fourth, data on ER expression were missing for a subset of patients (*n* = 34, 5.6%), and the analysis was performed as a complete-case analysis, excluding these cases. The potential impact of this missing data on the results should be considered a limitation of our study. Finally, data on other potential biomarkers (e.g., ESR1 mutations and Ki-67 index) were not available for the analysis.

We have refined our language to frame the strong recommendation for first-line use as a direct conclusion derived from the powerful effect of “naivety” observed in our data, which is intrinsically linked to the line of therapy.

We believe these clarifications have resolved the potential contradiction and provide a much clearer context for interpreting our results.

## 5. Conclusions

In conclusion, our real-world analysis identified treatment naivety and exceptionally high ER expression (≥90%) as key independent predictors of prolonged OS in HR-positive/HER2-negative metastatic breast cancer patients treated with CDK4/6 inhibitors. These findings underscore the importance of early CDK4/6 inhibitor implementation and suggest that quantitative ER assessment may refine patient selection beyond conventional positivity thresholds. The proposed ≥90% threshold warrants validation in prospective studies and collaborative pooled analyses to further personalise treatment strategies and optimise outcomes for patients with advanced breast cancer.

## Figures and Tables

**Table 1:** Patient Characteristics (*n* = 603).

Characteristic	N	%
Age (years)		
Median (Range)	56.5 (28–85)	
<35	29	4.8
35–50	186	30.8
51–65	220	36.5
>65	168	27.9
Menopausal Status		
Pre/Perimenopausal	201	33.3
Postmenopausal	401	66.5
Unknown	1	0.2
Tumor Characteristics		
Histological Subtype		
Invasive Ductal Carcinoma	525	87.1
Invasive Lobular Carcinoma	51	8.5
Other	19	3.2
Unknown	8	1.2
Receptor Status		
ER Expression		
<90%	259	43.0
≥90%	310	51.4
Unknown	34	5.6
PR Status		
Negative	139	23.1
Positive	430	71.3
Unknown	34	5.6
HER2 Status		
HER2-negative	301	50.0
HER2-low	297	49.3
Unknown	5	0.7
Disease Status		
Metastatic Presentation		
De novo	308	51.1
Recurrent	295	48.9
Metastatic Sites		
Visceral Metastasis	316	52.4
Liver Metastasis	136	22.6
Bone-Only Metastasis	135	22.4
Unknown	16	2.6
Treatment Characteristics		
CDK4/6 Inhibitor		
Ribociclib	422	70.0
Palbociclib	181	30.0
Prior Therapy History		
Prior Endocrine Therapy	144	23.9
Prior Chemotherapy	181	30.0

**Table 2:** Univariate Analysis of Overall Survival.

Variable	Median OS (Months)	HR (95% CI)	*p*-Value
Prior endocrine therapy			
No	51.0	1.68 (1.32–2.14)	<0.001
Yes	33.3		
Liver metastases			
No	50.0	1.42 (1.06–1.90)	0.019
Yes	34.0		
Bone-only metastases			
No	40.5	0.67 (0.48–0.94)	0.022
Yes	57.7		
PR status			
Positive	50.0	1.35 (1.02–1.79)	0.037
Negative	36.0		
ER status			
≥90%	49.0	1.24 (0.98–1.57)	0.072
<90%	41.0		
Prior chemotherapy			
No	47.0	1.38 (1.08–1.76)	0.010
Yes	38.5		
Visceral metastases			
No	51.0	1.34 (1.04–1.73)	0.025
Yes	40.5		

**Table 3:** Multivariate Cox Regression Analysis of Overall Survival.

Variable	HR	95% CI	*p*-Value
Prior endocrine therapy (Yes vs. No)	1.38	1.01–1.88	0.045
ER status (≥90% vs. <90%)	0.74	0.56–0.97	0.031
Liver metastases (Yes vs. No)	1.25	0.92–1.70	0.157
Bone-only metastases (Yes vs. No)	0.86	0.60–1.23	0.408
PR status (Negative vs. Positive)	1.22	0.89–1.66	0.216
Prior chemotherapy (Yes vs. No)	1.13	0.86–1.49	0.380
Visceral metastases (Yes vs. No)	1.18	0.89–1.57	0.247

## Data Availability

The data presented in this study are available upon request from the corresponding author. The data are not publicly available due to privacy and ethical restrictions.
